# Immune Reconstitution and Graft-Versus-Host Reactions in Rat Models of Allogeneic Hematopoietic Cell Transplantation

**DOI:** 10.3389/fimmu.2012.00355

**Published:** 2012-11-30

**Authors:** Severin Zinöcker, Ralf Dressel, Xiao-Nong Wang, Anne M. Dickinson, Bent Rolstad

**Affiliations:** ^1^Department of Anatomy, Institute of Basic Medical Sciences, University of OsloOslo, Norway; ^2^Department of Immunology, Oslo University Hospital – RikshospitaletOslo, Norway; ^3^Department of Cellular and Molecular Immunology, University Medical Center GöttingenGöttingen, Germany; ^4^Department of Haematological Sciences, Institute of Cellular Medicine, Medical School, Newcastle UniversityNewcastle-upon-Tyne, UK

**Keywords:** hematopoietic stem cell transplantation, graft-versus-host disease, rodentia, animal models, skin explant assay

## Abstract

Allogeneic hematopoietic cell transplantation (alloHCT) extends the lives of thousands of patients who would otherwise succumb to hematopoietic malignancies such as leukemias and lymphomas, aplastic anemia, and disorders of the immune system. In alloHCT, different immune cell types mediate beneficial graft-versus-tumor (GvT) effects, regulate detrimental graft-versus-host disease (GvHD), and are required for protection against infections. Today, the “good” (GvT effector cells and memory cells conferring protection) cannot be easily separated from the “bad” (GvHD-causing cells), and alloHCT remains a hazardous medical modality. The transplantation of hematopoietic stem cells into an immunosuppressed patient creates a delicate environment for the reconstitution of donor blood and immune cells in co-existence with host cells. Immunological reconstitution determines to a large extent the immune status of the allo-transplanted host against infections and the recurrence of cancer, and is critical for long-term protection and survival after clinical alloHCT. Animal models continue to be extremely valuable experimental tools that widen our understanding of, for example, the dynamics of post-transplant hematopoiesis and the complexity of immune reconstitution with multiple ways of interaction between host and donor cells. In this review, we discuss the rat as an experimental model of HCT between allogeneic individuals. We summarize our findings on lymphocyte reconstitution in transplanted rats and illustrate the disease pathology of this particular model. We also introduce the rat skin explant assay, a feasible alternative to *in vivo* transplantation studies. The skin explant assay can be used to elucidate the biology of graft-versus-host reactions, which are known to have a major impact on immune reconstitution, and to perform genome-wide gene expression studies using controlled combinations of minor and major histocompatibility between the donor and the recipient.

## Hematopoietic Cell Transplantation in Humans and Animals

At present, approximately 25,000 allogeneic hematopoietic cell transplantations (alloHCT) are performed each year worldwide, and the total number of such clinical procedures is predicted to rise in the future (Savani et al., 2011). AlloHCT can give rise to considerable toxicity and negative side-effects resulting in relatively high morbidity and mortality with poor quality of life and 5-year survival rates of 40–60% overall. It is however the only curative therapy in patients with relatively severe and advanced diseases for whom other treatment options are limited.

To date, alloHCT is complicated by opportunistic infections, disease relapse and graft-versus-host disease (GvHD). GvHD is an immunological condition that is caused by the reactivity of allogeneic T cells transferred in the stem cell graft from a genetically related or unrelated donor. After activation through recognition of disparate host antigens, alloreactive cells exert pathological damage via cytotoxic mechanisms and cytokine release on various organs and tissues, primarily the liver, gut, and skin, of the immunocompromised transplant recipient (Ferrara et al., 2008). In the clinical setting, relatives are favored over unrelated volunteer stem cell donors because of genetic similarity with the patient, reducing the overall risk of transplant failure and adverse graft-versus-host reactions (GvHR).

Immunity is deficient after alloHCT due to underlying disease, pre-transplant conditioning, or both, and it takes weeks to several months or years for the patient’s immune system to recover (Storek, 2008; Seggewiss and Einsele, 2010; Bosch et al., 2012). Early after transplantation, the reconstitution of immune cells in lymphoid organs is inhibited: the primary sites of white and red blood cell generation, bone marrow and thymus, are damaged by chemotherapy and radiation conditioning, and they are targeted by GvHD (Krenger and Holländer, 2008; Shono et al., 2010). Furthermore, immune function is impaired in elderly patients (Krenger et al., 2011) as both the regeneration of hematopoietic cells in the bone marrow and the thymic output of T lymphocytes decrease with age (Dorshkind et al., 2009).

Deficient and delayed restoration of immunity post-transplant contributes to the patient’s susceptibility to opportunistic infections, recurrence of the original disease and occurrence of secondary malignancies (Toubert et al., 2012). The individual contributions of natural killer (NK) cells, CD8^+^ cytotoxic T cells, CD4^+^ helper T cells, B cells, and innate immune cells, and their combined effects on these complications are still not fully understood (Geddes and Storek, 2007; Bosch et al., 2012).

Moreover, T cell depletion of the graft, when applied to reduce the risk of GvHD and essential in haploidentical alloHCT between family members (Kolb, 2008), compromises immune reconstitution, illustrating that grafted “passenger” T cells are needed for successful engraftment. Depletion of T cells also abrogates the graft-versus-tumor (GvT) effect, as GvH and GvT effects are strongly correlated and transplanted donor T cells can either mediate beneficial GvT effects or cause GvHD (Appelbaum, 2001). The crucial question of how to avoid detrimental GvHR without compromising the curative potential of alloHCT through a strong GvT effect has yet to be solved by clinical hematologists and HCT researchers.

### The usefulness of animal models in studying hematopoietic cell transplantation and graft-versus-host disease

The wish to better understand the dynamic biological processes between graft and host following transplantation, with the overall aim to improve the clinical management of alloHCT, has driven research in the development of adequate laboratory methods and animal models. The use of such models in transplantation research has contributed substantially to our current understanding of the nature of GvHR and its underlying principles in GvHD (Welniak et al., 2007; Socié and Blazar, 2009). Experimental alloHCT using mice and dogs have been particularly instrumental for the discovery of a variety of new therapies and for testing their feasibility, toxicity, and efficacy (Ferrara et al., 2008; Socié and Blazar, 2009).

Inbred rodent strains can be systematically established and maintained, yielding genetically homogeneous and biologically well-characterized strains for experimentation. Experiments in live animals facilitate and accelerate, for example, the study of genetic effects and mapping of gene associations with observable pathological phenotypes. An array of murine experimental models that employ donor and host strain combinations designed for mismatches only in the major histocompatibility complex (MHC) gene region (*H-2* in the mouse), only in minor histocompatibility antigens, or both, are available for the study of immune reconstitution and GvHD (Schroeder and DiPersio, 2011). Fully incompatible strain combinations, such as the popular [C57BL/6 (*H-2^b^*) → Balb/C (*H-2^d^*)] mouse model, are most often applied. Combinations of mice strains that are MHC matched but differ in minor histocompatibility antigens, for example [B10.D2 (*H-2^d^*) → Balb/C (*H-2^d^*)], [DBA/2(*H-2^d^*) → B10.D2 (*H-2^d^*)], or [C3H.SW(*H-2^b^*) → C57BL/6 (*H-2^b^*)] transplantation models (Korngold and Sprent, 1987), can mimic fully human leukocyte antigen (HLA) matched HCT between unrelated individuals, but not semi-HLA compatible HCT between sibling pairs. The latter scenario of haploidentical HCT between first-degree relatives can be modeled by inter-crossing two inbred strains and using the resulting hybrid progeny of the F_1_ generation as recipients of a transfusion of parental leukocytes in so-called parent-into-F_1_ models.

The mouse is historically and presently the most widely used model in biomedical research. However, other animal models have the potential to complement, validate, and extend the findings from experiments on mice. Studies of alloHCT in dogs make a case in point as they have added important knowledge in this field of research (Storb and Thomas, 1985), e.g., the use of delayed donor lymphocyte infusions to induce a GvT reaction and non-myeloablative (“reduced intensity”) conditioning regimens to reduce toxicity and morbidity in patients. Murine models have been employed to test non-myeloablative conditioning regimens with reduced irradiation intensity and/or combination chemotherapy (fludarabine, busulfan, cyclophosphamide, etc.; Santos and Owens, 1969; Ruggeri et al., 2002; Sadeghi et al., 2008). Similarly, rat alloHCT models based on chemotherapeutic conditioning without the need for irradiation have been developed (Santos and Owens, 1966; Tutschka and Santos, 1975; Okayama et al., 2004).

Rats offer all the advantages of a rodent experimental model, such as short generation time and cost effectiveness. Rats are bigger than mice (about 10-fold in body weight) and thus yield more grams of biological material per animal and per cage unit that can be sampled in the course of experimentation. Their spleens, lymph nodes, efferent lymph, and peripheral blood contain lymphocytes that can be readily harvested and serve as organ sources of donor grafts for transplantation. Hematopoietic stem cells are commonly obtained from the marrow of large bones (e.g., femurs and tibiae). Cell grafts enriched in Sca-1^+^ and CD34^+^ hematopoietic stem cells that are being used in patients and experimentally in mice have not been available for rat models due to the lack of specific antibody markers for targeted cell purification.

Many genetically defined and well-characterized inbred rat strains are available for research in transplantation immunology, and a range of rat alloHCT models have been developed from these strains during the past decades. Especially the strains *Brown-Norway* (BN) and *Lewis* (LEW) are widely used for fully MHC mismatched alloHCT (Santos and Owens, 1966; Clancy et al., 1976; Pakkala et al., 2001; Okayama et al., 2004; Zhu et al., 2011; Lin et al., 2012). Also HCT between haploidentical parental and filial generations, e.g., transplantation of LEW or BN bone marrow into F_1_ (BN × LEW) recipients, has been modeled in the rat (Clancy et al., 1983; Kimura et al., 1995; Ohajekwe et al., 1995; Peszkowski et al., 1996; Vaidya et al., 1996; Goral et al., 1998; Kobayashi et al., 1998; Sasatomi et al., 2005; Wolff et al., 2006; Kitazawa et al., 2012). In a number of these models, engraftment, reconstitution, chimerism, cell trafficking, and tolerance toward donor cells has been studied (Clancy et al., 1983; Oaks and Cramer, 1985; Ohajekwe et al., 1995; Engh et al., 2001; Foster et al., 2001; Okayama et al., 2004; Itakura et al., 2007; Klimczak et al., 2007; Nestvold et al., 2008; Zhou et al., 2008; Zhu et al., 2011; Zinöcker et al., 2011a;Lin et al., 2012). Furthermore, rat models have been employed to test prevention or treatment of GvHD by therapeutic regimens involving immunomodulatory drugs (Tutschka et al., 1979; Vogelsang et al., 1986; Vogelsang et al., 1988; Mrowka et al., 1994; Ohajekwe et al., 1995; Pakkala et al., 2001; Okayama et al., 2006; Wolff et al., 2006; Jäger et al., 2007), infusion or induction of various suppressive cell types (Itakura et al., 2007; Aksu et al., 2008; Nestvold et al., 2008; Kitazawa et al., 2010; Zinöcker et al., 2011b; Kitazawa et al., 2012; Zinöcker et al., 2012), UV irradiation (Ohajekwe et al., 1995; Gowing et al., 1998), serum transfusion (Shimizu et al., 1997), surgical techniques (Kobayashi et al., 1998), and prolonged distribution of a chemical agent with subcutaneously implanted osmotic pumps (Fidler et al., 1993).

The MHC is the dominant genomic region that governs mutual tolerance, rejection, and GvHR between the donor and the host in alloHCT. The mouse and rat MHC regions are closely related and share overall similarity with the human MHC (*HLA*; Kelley et al., 2005a), however, some important structural differences exist. In particular, the rat MHC (*RT1*) differs from the mouse MHC region in that it encompasses a large number of polymorphic non-classical MHC class I genes which serve as potential ligands for NK cell receptors (Vance et al., 1998; Naper et al., 2002; Naper et al., 2005; Kveberg et al., 2010; Andrews et al., 2012). In addition, the rat genome encodes a broader spectrum of inhibitory and activating Ly49 receptors expressed by NK cells compared to the mouse (Kelley et al., 2005b). Therefore, alloreactive rat NK cells and NK cell subsets may behave differently than mouse NK cells in the context of transplantation. Transplanted donor-versus-recipient NK cells mediate both GvT and GvH effects in patients (Ruggeri et al., 2002). They are thus a key element in the risk of leukemia relapse after haploidentical alloHCT (Velardi, 2012) and could influence T cell alloreactivity and the development of GvHR. The considerable differences especially in the NK cell system even between closely related species like rat and mouse illustrate the importance of relying on more than one experimental animal model in HCT research.

The complete genome sequence of *Rattus norvegicus* has been resolved in 2004 (Rat Genome Sequencing Project Consortium, 2004). With the advent of commercial cloning technology for rats (Huang et al., 2011) this species will likely be applied more frequently as a study object in the future. In the following sections, we will discuss some contributions by which rat models have helped to advance our understanding of immune reconstitution and GvHR following alloHCT.

### The rat as an *in vivo* model of immune reconstitution and graft-versus-host reactions after hematopoietic cell transplantation

To study the role of GvHR on immune reconstitution *in vivo*, we employed experimental transplantation protocols using combinations of rat strains with defined genetic incompatibilities (Engh et al., 2001; Nestvold et al., 2008; Zinöcker et al., 2012). The genetic composition regarding the MHC region of relevant rat strains are listed in Table 1. For experiments on fully incompatible (mismatched at the MHC region as well as non-MHC genes) alloHCT, we employed a model using *Piebald Virol Glaxo* (PVG) rats as the donor strain and BN rats as recipients of transplants. Conveniently, the genetic make-up of the donor PVG.7B strain encodes an allele (the RT7.2 allotype) of the CD45 gene (Kampinga et al., 1990), which facilitates later detection of donor-derived cells distinguishable from host-derived cells by flow cytometry using allotype-specific antibody. The genetic background of the PVG.7B strain is identical with the original PVG strain; both carry the *c* haplotype of the rat MHC, i.e., *RT1^c^* (Table 1). The recipient BN strain has a different genetic background and carries the *n* haplotype of *RT1*, *RT1^n^*.

**Table 1:** **MHC haplotypes of inbred congenic and recombinant rat strains**.

Rat strain	MHC subregion		
	Classical class I	Class II	Non-classical class I
	*RT1-A*	*RT1-B/D*	*RT1-CE/N/M*
BN	*n*	*n*	*n*
LEW	*l*	*l*	*l*
LEW.1N	*n*	*n*	*n*
PVG	*c*	*c*	*c*
PVG.7B	*c*	*c*	*c*
PVG.1N	*n*	*n*	*n*
PVG.1U	*u*	*u*	*u*
PVG.R23	*u*	*a*	*av1*

The reconstitution of blood and immune cells, especially of T lymphocytes, has been extensively studied in patients (Holland et al., 2008; Toubert et al., 2012), however, the mechanisms behind reconstitution and immunodeficiency and the complex interactions between donor and host cell types in this context remain incompletely understood. Many useful insights into the underlying cellular and molecular processes have been gained from experiments done mainly in mice. Immune reconstitution and post-transplant immune responses have been characterized also in the laboratory rat to some detail, facilitating the design of new transplantation protocols (Nestvold et al., 2008). The main rat blood cell types and cell subsets with relevance to HCT and their standard phenotypic characterization are summarized in Table 2.

**Table 2:** **Immunophenotype of rat leukocyte populations**.

Cell type	Phenotype	Antibody	Reference
Leukocyte	CD45^+^	OX-1	Sunderland et al. (1979)
	MHC-I^+^	OX-18	Fukumoto et al. (1982)
T lymphocyte	CD2^+^	OX-34	Jefferies et al. (1985)
	CD3^+^	G4.18	Nicolls et al. (1993)
	CD5^+^	OX-19	Dallman et al. (1984)
	CD6^+^	OX-52	Castro et al. (2003)
αβ T cell	TCRαβ^+^	R73	Hünig et al. (1989)
CD4 αβ T cell	CD4^+^	W3/25	Williams et al. (1977)
		OX-38	Jefferies et al. (1985)
CD8 αβ T cell	CD8α^+^	OX-8	Brideau et al. (1980)
Naive T cell	CD25^–^	OX-39	Paterson et al. (1987)
	CD45RC*^hi^*	OX-22	Spickett et al. (1983)
T blast	CD25^+^	OX-39	Paterson et al. (1987)
CD4 T blast	CD134^+^	OX-40	Paterson et al. (1987)
Memory T cell	CD62L^–^	OX-85	Seddon et al. (1996)
	CD45RC*^lo^*	OX-22	Spickett et al. (1983)
Regulatory	CD45RC*^lo^*	OX-22	Powrie and Mason (1990)
CD4 T cell	CD25^+^	OX-39	Stephens and Mason (2000)
	FoxP3^+^	FJK-16s	Beyersdorf et al. (2005)
Regulatory	CD45RC*^lo^*	OX-22	Xystrakis et al. (2004b)
CD8 T cell	FoxP3^+^	FJK-16s	Han et al. (2007)
γδ T cell	TCRγδ^+^	V65	Kühnlein et al. (1994)
B lymphocyte	CD45RA^+^	OX-33	Woollett et al. (1985)
	MHC-II^+^	OX-6	McMaster and Williams (1979)
Activated B cell	CD80^+^	3H5	Maeda et al. (1997)
	CD86^+^	24F	Maeda et al. (1997)
NK lymphocyte	CD3^–^	G4.18	Nicolls et al. (1993)
	CD161a^+^	3.2.3	Na et al. (1992)
	(NKR-P1A)	10/78	Chambers et al. (1992)
NKT lymphocyte	CD3^+^	G4.18	Nicolls et al. (1993)
	CD161a^+^	3.2.3	Na et al. (1992)
	(NKR-P1A)	10/78	Chambers et al. (1992)
Macrophage	MHC-II^+^	OX-6	McMaster and Williams (1979)
	CD172a^+^ (SIRPα)	OX-41	Robinson et al. (1986)
	CD11c^+^	OX-42	Robinson et al. (1986)
	CD68^+^	ED1	Dijkstra et al. (1985)
		ED2^a^	Dijkstra et al. (1985)
Dendritic cell	MHC-II^+^	OX-6	McMaster and Williams (1979)
	CD172a^+^ (SIRPα)	OX-41	Robinson et al. (1986)
	CD11c^+^	OX-42	Robinson et al. (1986)
	α_E2_ integrin	OX-62	Brenan and Puklavec (1992)

Phenotypic markers of rat leukocyte populations and subpopulations are summarized together with available antibodies commonly used for their flow cytometric detection. Comprehensive lists of rat antigens and specific antibodies have been published by Puklavec and Barclay (2001) and van den Berg et al. (2001). A list of OX antibodies can be found online at http://users.path.ox.ac.uk/~ciu/mrc-mabs.html

*^a^No CD designation is assigned for the cognate antigen*.

**Figure 1:**
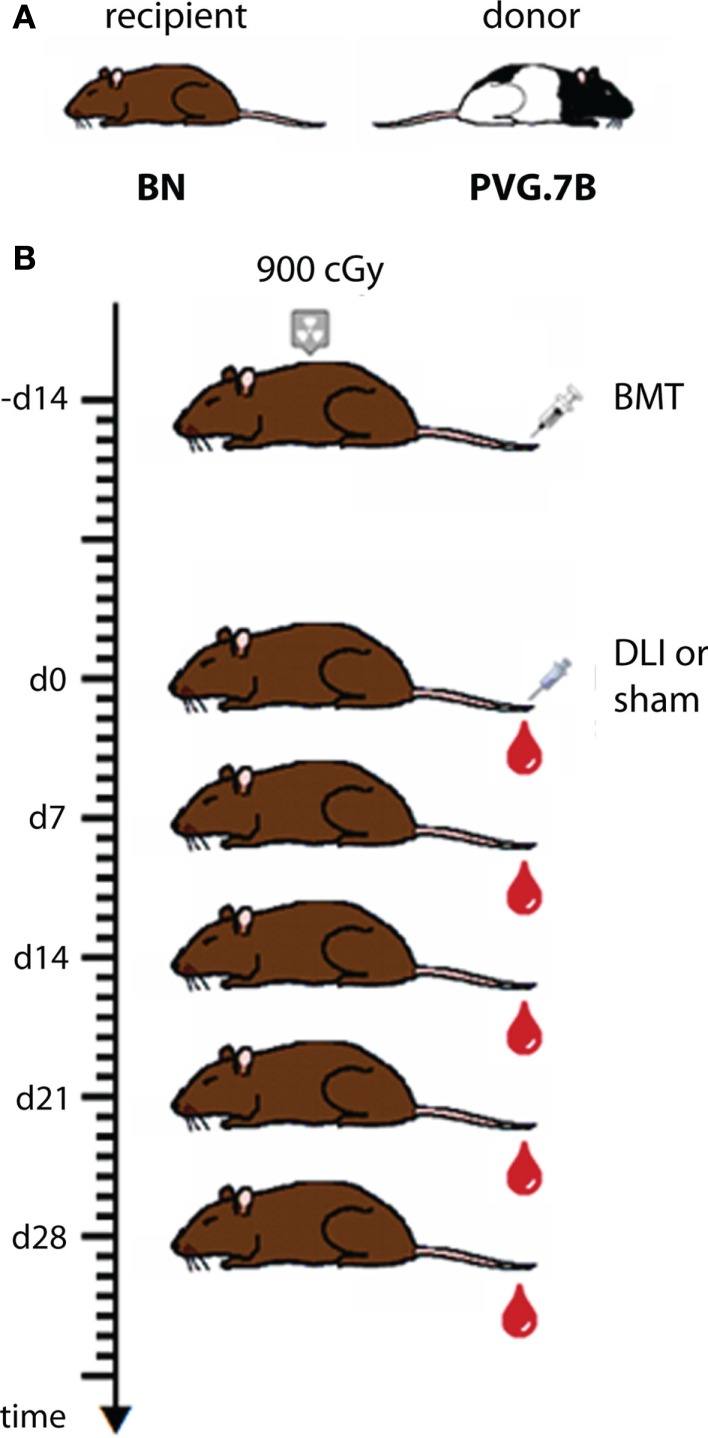
**Schematic outline of the transplantation protocol applied in the MHC mismatched rat model. (A)** BN rats served as recipients and PVG.7B rats as bone marrow and lymph node donors for bone marrow transplantation (BMT) and donor lymphocyte infusion (DLI), respectively, in fully MHC-incompatible alloHCT performed as shown in **(B)**. Recipient rats were lethally irradiated (900 cGy), rescued by an intravenous injection of donor bone marrow cells and 14 days later received a graded dose of donor lymph node cells to invoke fatal GvHD. Controls received sham treatment (injection without cells) instead of DLI. Blood samples were collected in weekly intervals from the day of DLI for subsequent analysis of reconstitution and chimerism.

One important aim of our studies was to describe in detail the reconstitution of the lymphocytes that mediate GvT and GvH effects, i.e., T cells and NK cells, in bone marrow recipients and in experimental GvHD. The experimental protocol used in these studies (Zinöcker et al., 2011a; adapted from Nestvold et al., 2008) is schematically outlined in Figure 1. Recipient rats received a lethal dose of 900 cGy total body irradiation from a Cesium-137 source emitting β and γ irradiation at an approximate rate of 4 Gy min^–1^. This conditioning regimen made recipient rats susceptible to donor engraftment. Donor bone marrow containing hematopoietic stem cells was obtained from leg bones (femurs and tibiae). The graft consisted of mononuclear bone marrow cells that were depleted of T cells by negative selection using magnetic beads with anti-αβTCR and anti-CD5 antibodies (cf. Table 2) prior to transplant (Nestvold et al., 2008). Thus, the primary graft served to establish stable donor engraftment. In this model, the transfer of donor-versus-recipient reactive NK cells (Løvik et al., 1995) contained in the graft should facilitate engraftment and donor reconstitution. GvT effects can be studied after injecting BN acute myelocytic leukemia cells, a model of human acute myeloid leukemia (Martens et al., 1990), into BN recipients (Nestvold et al., 2008). Transplanted rats were convalescent for 14 days before GvHD was induced in a cell-dose dependent manner by a single infusion of donor lymphocytes (DLI; cf. Figure 1) from lymph nodes. Alternatively, GvHD can be invoked by transfusion of donor cells derived from the spleen or peripheral blood, and the latter source is highly relevant with regard to clinical GvHD, which is commonly caused by the transfer of T cells contained in a donor blood graft or donor lymphocyte transfusion.

#### T cells, NK cells, and NKT cells

With respect to lymphocyte reconstitution in recipients of clinical HCT, NK cells recover relatively swiftly (weeks) and T cell reconstitution happens more slowly (months to years; Storek et al., 2008). In our hands, both NKT and NK cells (as defined by CD3 and NKR-P1 surface expression; cf. Table 2) of donor origin were rapidly generated in animals transplanted with T cell-depleted bone marrow. NKT and NK cells of host origin were not detected in the circulation 2 weeks after HCT (Zinöcker et al., 2011a). Donor T cells showed a significantly slower pace of reconstitution in these animals, and reached approximately 90% donor chimerism in the blood only several months after transplant (Zinöcker et al., 2011a). Thus, the overall patterns of NK, NKT, and T lymphocyte reconstitution were similar to those observed in patients, albeit with accelerated kinetics as might be expected from a relatively short-lived rodent species.

Donor CD4^+^ T cells showed a tendency to reconstitute slightly more slowly than did donor CD8^+^ T cells (Zinöcker et al., 2011a), which is also comparable with known clinical data (Seggewiss and Einsele, 2010). Rats that received an infusion of donor lymph node cells and subsequently developed GvHD displayed full donor chimerism of CD4^+^ and CD8^+^ T cells shortly after DLI, coinciding with the onset of disease symptoms. This increase very likely reflected the activation by allo-antigens and rapid expansion of donor CD4^+^ and CD8^+^ T cell clones in the host. Any remaining host T cells were probably cleared by alloreactive donor cells.

#### Memory T cells

We also noted that donor-derived CD62L-expressing naive T cells were less frequent in the peripheral blood of rats that developed GvHD compared with healthy bone marrow-transplanted controls (Zinöcker et al., 2011a). This finding may likely be due to the loss of CD62L surface expression in the course of T cell activation as measured by the concomitant increase of CD25 and CD134 activation markers on CD4^+^ and CD8^+^ T cell populations (cf. Table 2). Although we did not specifically investigate a memory T cell phenotype in our study, we speculate that donor memory T cells may expand in the presence of host allo-antigens and allo-activation as CD62L^−^ T cells were more frequent in the circulation in rats with GvHD. CD44^+^ CD62L^−^ memory T cells that are generated in response to pathogens during infection as well as other antigens have been reported not to cause GvHD (Anderson et al., 2003; Chen et al., 2004). In line with these studies, Xystrakis et al. (2004a) have employed a parent-into-F_1_ HCT model and showed that CD4^+^ CD45RC*^lo^* T cells containing mainly memory T cells, in contrast to CD4^+^ CD45RC*^hi^* T cells containing mainly naive T cells, failed to induce acute or chronic GvHD in recipient rats. Conversely, transplanted memory T cells not specifically primed to allo-antigens can have a considerable potency to induce GvHD (Zheng et al., 2009). Therefore, the capacity of differentiated memory T cells to elicit a GvHR may depend on the nature of their primary antigen encounter.

#### Regulatory T cells

Furthermore, we found that host-derived FoxP3-expressing CD4^+^ CD25^+^ T cells, comprising a regulatory T cell subtype (Beyersdorf et al., 2005), reconstituted relatively quickly and were present at normal levels in peripheral blood at 4 weeks post-transplant (Zinöcker et al., 2011a). It has been shown previously in mice that recipient CD4^+^ CD25^+^ T cells can resist irradiation and regulate chronic GvHD (Anderson et al., 2004). Donor-type FoxP3^+^ regulatory T cells, on the other hand, did not amount to significant numbers in the blood of transplanted rats at any time point post-transplant. Strikingly, and in contrast to transplanted controls which did not receive DLI, rats that suffered from GvHD failed to reconstitute FoxP3^+^ regulatory T cells completely. We speculate that the absence of regulatory T cells is a major contributing factor in the lack of ability of these animals to avert alloreactivity as they uniformly developed lethal GvHD. Another regulatory T cell subtype that was defined as CD8^+^ CD45RC*^lo^* FoxP3^+^ in the rat (cf. Table 2) suppressed alloreactive donor CD4^+^ T cells *in vitro* and prevented experimental GvHD *in vivo* (Xystrakis et al., 2004b). Decreased FoxP3 expression (Miura et al., 2004) and frequency of regulatory T cells (Zorn et al., 2005; Rieger et al., 2006; Magenau et al., 2010) has been associated with the incidence of GvHD in patients. Furthermore, CD4^+^ CD25^+^ regulatory T cells have been shown to suppress host-reactive T cells and reduce acute GvHD in murine models (Cohen et al., 2002; Hoffmann et al., 2002; Taylor et al., 2002; Edinger et al., 2003). Alleviation of GvHD after infusion of donor-derived CD4^+^ CD25^+^ regulatory T cells has recently been reported in some patients (Brunstein et al., 2011; Di Ianni et al., 2011), although these phase I clinical trials were not powered to assess the efficacy of this treatment modality and did not include control subjects. The therapeutic potential of regulatory T cell induction or adoptive transfer in GvHD prevention is currently under clinical exploration (Leventhal et al., 2012).

#### B cells and antibodies

In transplanted rats, B cells recover gradually in host lymphoid organs and peripheral blood with kinetics similar to that of T cells (Renkonen, 1986). This is consistent with what is known of B cell reconstitution in patients who receive an allogeneic donor transplant (Bosch et al., 2012), as is the finding that B cell reconstitution is delayed during acute GvHD (Renkonen, 1986; Bosch et al., 2012). B cells did not recover in the skin, lungs, or the gut of host rats that developed acute GvHD, but accumulation of immunoglobulin-producing cells was observed in the inflamed liver (Renkonen et al., 1986). B cells are thought to participate in the pathogenesis of GvHD (Shimabukuro-Vornhagen et al., 2009), possibly by presenting allo-antigens and by secreting auto- and alloreactive antibodies.

Disparate MHC molecules and minor histocompatibility (non-MHC) antigens of both donor and host type co-exist in radiation chimeras, by which B cells can be activated to produce allo-antibodies either against donor antigens (on hematopoietic cells) or against host antigens (mainly non-hematopoietic tissue). Putative donor-derived allo-antibodies will therefore bind mainly to tissue components of the host and may be difficult to detect in the serum unless circulating donor cells are screened for B cell receptor specificities. We know that the transfusion of PVG T cells into semi-allogeneic F_1_ (PVG × DA) hybrid recipients induces auto-antibodies against basal membranes of the skin (Rolstad, 1985). Increased serum levels of antibodies against heat-shock proteins are associated with acute and chronic GvHD (Goral et al., 1995, 2002).

B cells play an important role in the etiology of chronic GvHD as increased auto-antibody titers and other autoimmune-typical symptoms are hallmarks of this disease (Ferrara et al., 2008). Laboratory rodents have been used to model chronic GvHD experimentally (Vogelsang et al., 1988; Schroeder and DiPersio, 2011). Spleen cell transfer without bone marrow transplantation from either BN or LEW donor strains induced autoreactive antibodies that are typical of an autoimmune-like chronic GvHR in F_1_ (LEW × BN) hybrid rats (Tournade et al., 1990; Xystrakis et al., 2004a). Interestingly, transfusions of LEW splenocytes resulted in acute GvHD and death of F_1_ recipients, while transfusions of BN splenocytes caused no disease (Tournade et al., 1990; Xystrakis et al., 2004a). This may be due to different genetic factors in the respective donor strains and T cell responses that differ in the pattern of cytokine release upon allostimulation (Bernard et al., 2010). Studies done in our labs support the hypothesis that the quality of the allogeneic T cell response is important and depended on the tissue source: peripheral T cells typically induced acute GvHD (Nestvold et al., 2008) while bone marrow-derived T cells induced a more chronic form of the disease (Naper et al., 2010).

### Allo-transplantation in a semi-compatible rat model

Hematopoietic cell transplantations using fully MHC mismatched donors are not performed in the clinic because of the excessively high risk of GvHD. Previous efforts in our lab to generate a semi-compatible (haploidentical) HCT model by cross-breeding inbred PVG and BN rats failed because of what appeared to be a significant heterosis effect that rendered the F_1_ (PVG × BN) offspring highly aggressive and impossible to handle. In another effort to design a model that reflects the clinical reality better than the completely mismatched [PVG.7B → BN] transplantation model, we selected rat strains that were partly matched at the MHC region. The PVG-*RT1^r23^* strain (PVG.R23) and the PVG-*RT1^u^*strain (PVG.1U) are partially compatible: PVG.1U rats express the *u*-*u*-*u* haplotype of *RT1* and PVG.R23 rats the intra-MHC recombinant *u*-*a*-*av1* haplotype, i.e., they are matched in the classical class I (*RT1-A*), but not the class II (*RT1-B/D*) and non-classical class I (*RT1-CE/N/M*) MHC regions (cf. Table 1). The strains are matched for minor histocompatibility antigens as both express the same class III MHC genes on the PVG genetic background. Disparity for either the class II locus *RT1-B/D* or non-MHC genes elicits fatal GvHD in the rat, while the class I loci *RT1-A* and *RT1-CE/N/M*, respectively, have little or no effect (Oaks and Cramer, 1985). Therefore, in the [PVG.1U → PVG.R23] combination, GvHD is primarily due to the mismatch of class II genes. Dahlke and coworkers (Jäger et al., 2007) have made use of a similar semi-allogeneic transplantation model, where a mismatch only in MHC class II genes induced lethal GvHD in [LEW.AR1 → LEW.AR2] transplanted rats. We used PVG.R23 as HCT recipients and PVG.1U rats as marrow donors, respectively, in our experiments (Zinöcker et al., 2012). This model was designed to better mimic a clinical setting where some degree of mismatch of the donor MHC is permissible to perform a HCT procedure on patients in need of a transplant (Petersdorf, 2007). It should not be inferred from this model, however, that it can replicate the setting of haploidentical HCT in humans, where siblings, who share half of their MHC genes and a range of minor histocompatibility antigens with the recipient, serve as stem cell donors. Rather, the semi-compatible rat HCT model reflects a transplantation scenario in which polymorphisms of both MHC and non-MHC genes contribute to the induction of acute GvHD.

The HCT protocols described above (cf. Figure 1) resulted in the onset of a rapid wasting disease with severe loss of weight that was typical of acute GvHD and was fatal in 90–100% of recipients (Zinöcker et al., 2011a,b, 2012). The kinetics of disease progression were similar in both the MHC mismatched [PVG.7B → BN] and the partially matched [PVG.1U → PVG.R23] model (Zinöcker et al., 2012). The titrated DLI dose required to reproducibly invoke lethal acute GvHD in the latter model (9 × 10^6^ T cells from lymph nodes) was higher compared to the former (5 × 10^6^ T cells from lymph nodes; Zinöcker et al., 2012). Recipients were monitored closely over time for GvHD-typical symptoms which were quantified using a scoring table (Zinöcker et al., 2011a) adapted from Cooke et al. (1996).

**Figure 2:**
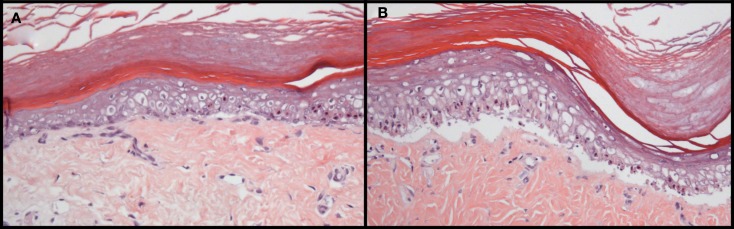
**Histopathology of GvHD in rats transplanted with allogeneic, fully MHC mismatched bone marrow**. Grade II **(A)** and III–IV **(B)** pathology is manifest in the skin of BN rats that suffered from acute GvHD after bone marrow transplantation and donor lymphocyte infusion from PVG donors. Pathological grading was performed based on the GvHD classification originally described by Lerner et al. (1974) for human skin GvHD. Skin was removed from the paws at autopsy and tissue sections were stained with hematoxylin and eosin. These photographs are reprinted from Novota et al. (2008) with permission from the publisher.

Acute GvHD primarily affects the liver, the intestine and the skin (Ferrara et al., 2008). In our models, severe GvHD symptoms were observed (Zinöcker et al., 2011a). The skin was targeted by GvHR; poor grooming, ruffling, fur loss, skin flaking, and occasionally, skin lesions were manifest. These observations were confirmed histologically, with GvHD symptoms present in the skin (Figure 2) and secondary lymphoid organs, spleen and lymph nodes (Zinöcker et al., 2011a). Histological evaluation of the liver and gastrointestinal tract revealed no significant pathological changes of these organs. Therefore, results from these models pertain primarily to cutaneous GvHD. The absence of observable pathology of the liver and the gut could simply depend on the rapid onset and progression of GvHD in host rats, marked by dehydration and severe weight loss, before histopathology of other organs became manifest. On the other hand, they may reflect significant differences in the underlying mechanisms of disease development affecting distinct target sites in these models.

Despite the considerable genetic barriers in the fully and partially mismatched alloHCT settings, we reproducibly established stable allogeneic rat chimeras by transplanting large numbers of bone marrow cells that were extensively depleted of T cells. These protocols demonstrate the central importance of T cells in GvHD as is the case in humans (Ferrara et al., 2008), and, as defined numbers of donor T cells were sufficient to subsequently induce severe acute GvHD, these models may serve to investigate the contributions of T cells and other cell types to GvHR *in vivo* and to manipulate such alloimmune responses with drugs or different modes of cellular therapy.

One important advantage of using the rat model, which we discuss further in the following section, is the availability of corresponding *in vitro* models of GvHR, especially the rat skin explant assay. This model resembles key features of clinical GvHR in a simple experimental setting that can be highly standardized and easily manipulated.

## The Rat Skin Explant Assay

Robust risk estimates of GvHD incidence and severity would allow clinicians to intervene at the stage of donor selection, pre-transplant conditioning, and choice of immunosuppressive treatment in the course of transplantation. It is well established that disparities in minor and major human leukocyte antigens between patients and donors can cause GvHD. A number of human biomarkers of acute and chronic GvHD for prediction of treatment outcome and mortality have been identified recently (Paczesny et al., 2009; Levine et al., 2012b) and their reliability was prospectively evaluated in patients (Levine et al., 2012a).

The skin explant assay is an *in vitro* model which can predict GvHD in patients with high sensitivity and specificity. It was prospectively tested as a method to predict GvHD incidence and severity in a donor/patient cohort by Vogelsang et al. (1985). Several studies used the skin explant assay to predict the likelihood of acquiring GvHD and reported correlation rates of 80–90% with clinical outcome, which proved the skin explant assay superior to other *in vitro* models that had been designed for this purpose (Sviland and Dickinson, 1999; Dickinson et al., 2001; Hromadnikova et al., 2001; Sviland et al., 2001; Wang et al., 2006). The accuracy of the prediction by skin explant, however, was dependent on the type of GvHD prophylaxis and conditioning regimens used. Matched unrelated donor transplants, including T cell depletion protocols, gave understandably low prediction rates (Wang et al., 2006).

**Figure 3:**
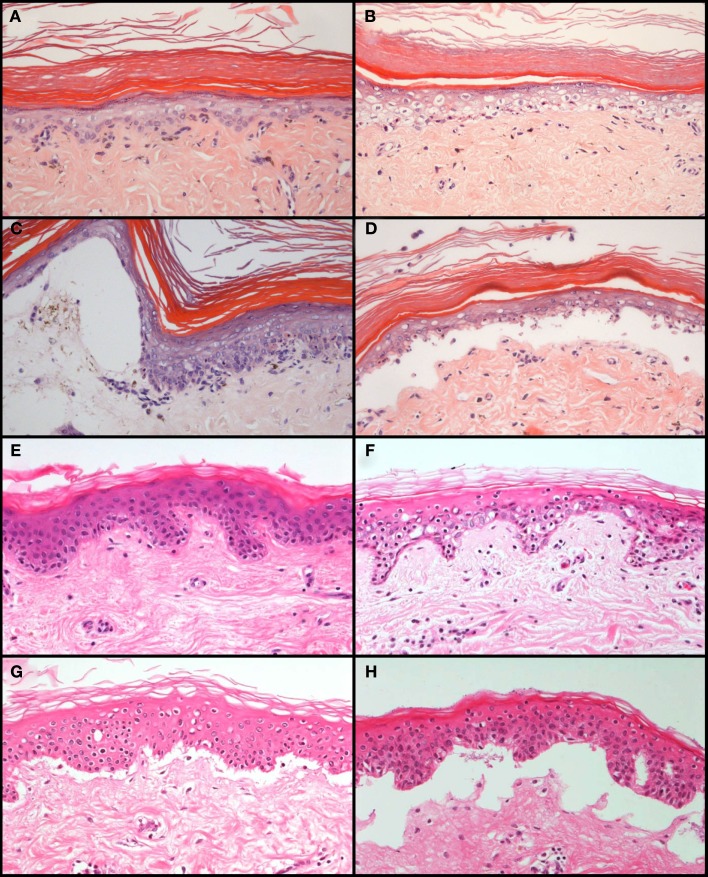
**Comparison of the histopathology of GvHR in human skin explants and rat skin explants**. GvHR of increasing severity occur during co-incubation of pre-stimulated, allogeneic lymphocytes with explants of rat skin **(A–D)** or human skin biopsies **(E–H)**. Grade I [mild vacuolization of epidermal cells with occasional dyskeratotic bodies; **(A,E)**], grade II [diffuse vacuolization of basal cells with scattered dyskeratotic bodies; **(B,F)**], grade III [formation of subepidermal clefts; **(C,G)**], and grade IV [complete separation of the epidermis from the dermis; **(D,H)**] pathological changes are similar in human and rat skin explants. The photographs of the rat skin explants depicted in **(A–D)** were reprinted from Novota et al. (2008) with permission from the publisher.

The skin explant closely resembles the histopathology of cutaneous GvHD and allows the study of pathophysiological processes related to GvHR (Sviland and Dickinson, 1999; Dickinson et al., 2001; Dickinson et al., 2002). Histological grading of the skin explant, from grade I to grade IV with increasing severity (Lerner et al., 1974), represents a standardized method to evaluate the risk for selected donor/recipient combinations before transplantation. One problem for a broad application of the assay in experimental studies is the shortage and genetic heterogeneity of skin biopsies from healthy donors. To overcome this problem and to reduce the need for animal experimentation, we developed a rat skin explant assay that can complement and in part replace transplantations in rodents. This assay can be highly standardized and results are not hampered by undefined genetic differences between tissue samples which cannot be avoided in human studies. The experimental procedure is described in detail elsewhere (Novota et al., 2008). Briefly, lymphocytes from the spleens of donors are stimulated with irradiated lymphocytes from the spleens of recipients in a mixed lymphocyte reaction for 7 days, then transferred to a cultured skin biopsy of the recipient and co-incubated for three more days. The GvHR is then evaluated and scored by histology using the same criteria as for grading GvHR in human skin explants (Sviland and Dickinson, 1999). Using genetically well-defined inbred strains, we showed that combinations with differences in major histocompatibility antigens usually led to grade III or IV GvHR (Novota et al., 2008). GvHR was on average less severe (grade II) in MHC congenic strain combinations which are only mismatched in minor histocompatibility antigens than in combinations with major or minor plus major histoincompatibility (Novota et al., 2008). The histopathology of cutaneous GvHR in these skin explants closely resembled the histopathology of GvHR in human skin explants (Figure 3). We were also interested to determine whether the rat skin explant assay could resemble, in addition to histological features of GvHD, the molecular profile of GvHD in the patient. In our initial study (Novota et al., 2008), we focused on MHC-linked *Hsp70* genes because the expression of stress-inducible heat shock protein 70 has been correlated with the GvHR grading in human skin explants (Jarvis et al., 2003). Indeed, the relative expression of the *Hsp70-1* (*Hspa1b*) and *Hsp70-2* (*Hspa1a*) genes correlated with the grade of GvHR in rat skin explants (Novota et al., 2008), indicating that certain molecular patterns of GvHR are conserved between human and rat skin explants.

This result encouraged us to use the rat skin explant assay for searching new genetic biomarkers of GvHR. In an expression profiling study with special emphasis on MHC and NK gene complex (NKC) genes, 11 MHC, 6 NKC, and 168 genes in other genomic regions were identified to be regulated during GvHR in rat skin explants (Novota et al., 2011). For MHC and NKC genes, the results were verified by real time PCR experiments on samples from other skin explant assays using different strain combinations, on skin with cutaneous GvHD from transplanted rats, and on samples from human skin explant assays. The genes that were confirmed to be regulated also in human skin explant samples with GvHR included *TAP1*, *PSMB8*, *C2*, *UBD*, and *OLR1* (Novota et al., 2011). These genes have known polymorphisms in humans and are therefore potential candidates for diagnostic biomarkers in the clinic.

The rat skin explant assay is a promising tool not only for the identification of candidate genes regulated in GvHR but also for the functional evaluation of GvHR in an experimental system. As with the human skin explant assay, it can be used to investigate the role of cytokines (Dickinson et al., 1991; Dickinson et al., 2002; Wang et al., 2002) and innate immunity in GvHR, e.g., heat-shock protein 70 (Jarvis et al., 2002, 2003), the roles of NK cells, dendritic cells (Wilson et al., 2009), regulatory T cells (Wang et al., 2009; Mavin et al., 2012), and subsets of T cells (Dickinson et al., 2002) as well as Fas expression (Ruffin et al., 2011).

## Conclusion

The laboratory rat is a useful biological model organism for the study of alloHCT, immune reconstitution, and transplant-related complications. While the mouse has found its place in the limelight of comparative and translational immunology, a steadily increasing number of insightful investigations in the rat have been undertaken. When compared to mice and other animal models, experiments in rats can add instrumental insights into the consistency or divergence of findings made in related mammalian species. We found that after transplantation, host rats reconstituted peripheral regulatory T cells of host but not donor origin, and were deficient of any such cells when suffering from severe acute GvHD.

Important aspects of alloHCT, such as GvHR and tolerance mechanisms that are highly relevant for immune reconstitution can now be addressed *ex vivo* in the rat skin explant model. Therefore, this assay represents a valuable and accessible research tool that complements the *in vivo* model. These research tools currently available in the rat model might also prove to be important for other areas of research.

## Conflict of Interest Statement

The authors declare that the research was conducted in the absence of any commercial or financial relationships that could be construed as a potential conflict of interest.
